# The Charged Superhydrophilic Polyelectrolyte/TiO_2_ Nanofiltration Membrane for Self-Cleaning and Separation Performance

**DOI:** 10.3390/membranes15060179

**Published:** 2025-06-12

**Authors:** Weiliang Gu, Lei Han, Ye Li, Jiayi Wang, Haihong Yan, Zhenping Qin, Hongxia Guo

**Affiliations:** 1 State Key Laboratory of Materials Low-Carbon Recycling, College of Materials Science and Engineering, Beijing University of Technology, Beijing 100124, China; guweiliang@emails.bjut.edu.cn (W.G.); limingye@emails.bjut.edu.cn (Y.L.); jiayiwang@emails.bjut.edu.cn (J.W.); zhenpingq@bjut.edu.cn (Z.Q.); 2 Beijing Nansheng Technology Co., Ltd., Beijing 102601, China; 3 Xinkai Environment Investment Co., Ltd., Beijing 101101, China; hanlei726@126.com; 4 State Key Laboratory of Environmental Criteria and Risk Assessment, Research Center of Environmental Pollution Control Engineering Technology, Chinese Research Academy of Environmental Sciences, Beijing 100012, China

**Keywords:** nanofiltration, negatively charged TiO_2_, photocatalysis, self-cleaning

## Abstract

Nanofiltration (NF) technology has extensive application in the treatment of wastewater generated in the dyeing industry. However, NF membranes often encounter fouling issues during the operation process. In this work, the superhydrophilic and self-cleaning multilayer nanofiltration membrane was prepared by self-assembling polyelectrolyte incorporating the anatase PSS-TiO_2_ nanoparticles. The negatively charged PSS-TiO_2_ nanoparticles were beneficial to the formation of the nanohybrid selective layers via electrostatic interforce. The prepared (PEI/PSS-TiO_2_)_4.0_ hybrid membrane showed favorable photoinduced superhydrophilicity. The water contact angle of the membrane decreased with the UV irradiation from 35.7° to 1.6°. The negatively charged (PEI/PSS-TiO_2_)_4.0_ membrane exhibited a 100% rejection rate to XO and EbT, with a permeance flux of 5.2 and 6.4 L/(m^2^·h·bar), respectively. After UV irradiation for 60 min, the permeance flux could be further increased to 13.4 and 14.0 L/(m^2^·h·bar), and the rejection remained at 97.8% and 96.7%. Owing to the low content of TiO_2_ NPs photocatalytic effect under UV irradiation, the fabricated hybrid membrane exhibited a compromised permeance recovery of about 80.6%.

## 1. Introduction

Nanofiltration (NF) is an effective pressure-driven technique and has extensive applications in various aqueous effluent treatments [1,2,3,4,5,6], such as water softening [1,2], industrial wastewater treatment [3,4], pharmaceuticals effluent discharge [5,6] and so on [7,8,9]. As the key to the NF system, the NF membrane has drawn growing attention due to its attractive benefits such as lower osmotic pressure, high retention, manageability and relatively low investment [1,8,9]. Nevertheless, the membrane fouling inevitably causes several negative effects on NF performance such as flux decline, membrane decay and an increase in operation and maintenance costs [9,10]. Designing NF membranes to balance antifouling resistance and high solute retention and permeance as well as improved stability remains the focus.

Various strategies have endeavored to improve the antifouling property of NF membranes, including manipulating the membrane surface to be more hydrophilic [11,12] and incorporating inorganic components, such as multiwalled carbon nanotube [13,14], silica [15,16], graphene oxide (GO) [17,18] and modified Mg(OH)_2_ nanostructures [19,20], into the active layer of thin-film nanocomposite nanofiltration (TFN–NF) membrane. Owing to nano-sized TiO_2_ nanoparticles displaying photoinduced superhydrophilic conversion under UV radiation, organic contaminants on its surface could be efficiently removed [21,22]. Then, TiO_2_ nanoparticles have been incorporated into membranes to endow self-cleaning/antifouling properties and enhance the permeability through photocatalytic and superhydrophilicity effects [23,24,25]. The traditional way to immobilize TiO_2_ is to blend the nanoparticles with casting solution to form membrane by phase inversion or coating method [26,27]. This method is easily carried out, but the effects of TiO_2_ nanoparticles on membrane morphology, especially on the porosity, roughness and surface hydrophilicity when they agglomerated, could not be ignored [28,29]. Moreover, the photodegradation rate might be suppressed due to the entrapped nanoparticles were not easily accessible to substrate molecules [30].

Gong et al. used the in situ growth of TiO_2_ nanoparticles in polyelectrolyte (PE) multilayers to improve the dispersibility and form the superhydrophilic membrane, which showed high loadings of inorganic components and avoided the excessive agglomeration of nanoparticles [31,32]. However, such a method is often induced in the amorphous TiO_2_ nanoparticles and obviously affects the photocatalysis of the membrane. To address these issues, other researchers employed the self-assembly method, wherein TiO_2_ was bound on the surface of the membrane through non-covalent interactions. The procedure was carried out by dipping the membrane in TiO_2_ suspension for a certain period to allow the deposition [25,33]. Yet, the challenge of this method is the weak interaction between the surface of the membrane and TiO_2_ nanoparticles. You et al. used plasma-grafting poly(acrylic acid) on commercial poly(vinylidene fluoride) membrane to introduce functional groups on the membrane surface that can support the nanoparticles [34]. Kim et al. prepared the composite membranes via self-assembly between TiO_2_ nanoparticles and carboxylic acid groups on the surface of the composite reverse osmosis membrane [35,36]. Shi et al. prepared PVDF/TiO_2_ hybrid microfiltration membranes with ionic liquid modified nano-TiO_2_ (IL-TiO_2_) via thermally induced phase separation (TIPS) method [37]. These methods were relatively complex and the functional groups on the surface of membrane/TiO_2_ may have effects on the hydrophilic and photocatalytic properties to some extent. Up to now, little information is available on the immobilization of charged TiO_2_ on nanofiltration membranes via static electrostatic self-assembled method, especially regarding their antifouling and photocatalytic performance. The positive and negative charged TiO_2_ nanoparticles can assemble on the membrane by the intercalation of cation and anion PEs. Furthermore, electro-conductivity is very important to the nanofiltration Donnan effect that prevent adsorption of particulate contaminant by an antistatic effect [38].

In this work, the highly negatively charged PE-TiO_2_ hybrid membrane is fabricated by incorporating the negatively charged anatase TiO_2_ nanoparticles into the self-assembled membrane. Such charged TiO_2_ was beneficial to subsequently assembly nanohybrid selective layers by an electrostatic interforce. The nanofiltration performance and the photocatalysis of the membrane were investigated. The nanohybrid membrane showed satisfactory nanofiltration for dye removals. The superhydrophilic self-cleaning membrane can be obtained via UV irradiation. This work provided a new strategy to manipulate the membrane surface charge and enhance the nanofiltration performance, making it a potential application in wastewater treatment.

## 2. Experimental Section

### 2.1. Materials

The polyacrylonitrile (PAN) ultrafiltration membrane with molecular weight cutoffs (MWCO) of 100 kDa was purchased from Sepro. Membranes Inc. (Oceanside, CA, USA). Before use, the PAN substrate membrane was hydrolyzed with 1.0 M NaOH solution for 1.0 h at room temperature to obtain a hydrolyzed PAN (hPAN) membrane, referencing the previous method [39]. It was then washed with DI water to achieve a neutral solution. Poly(ethyleneimine) (PEI, Mw = 750,000, 99.0% purity) and poly(sodium4-styrenesulfonate) (PSS, Mw = 70,000, 99.0% purity) were purchased from Sigma-Aldrich (St. Louis, MO, USA). Xylenol orange (XO, 99.0% purity) and eriochrome black T (EbT, 99.0% purity) dyes were provided by the Beijing Chemical Factory (Beijing, China). Titanium sulfate (TiOSO_4_, 97.0% purity), hexamethylenetetramine (C_6_H_12_N_4_, HMT, 99.0% purity), and nitric acid (HNO_3_, 99.7% purity) were provided by the Beijing Chemical Factory. Unless specifically stated, all chemicals were used without further purification.

### 2.2. Preparation of TiO_2_ Hybrid Membrane by Self-Assembly

Prior to the preparation of the PE-TiO_2_ hybrid membrane, the negatively charged PSS-TiO_2_ NPs were first prepared, which can be well dispersed in corresponding PSS solution. The PSS-modified anatase TiO_2_ NPs were prepared via a low-temperature precipitation–peptization process (LTPPP) according to our previous method [39]. Specifically, 4.6 wt% of TiOSO_4_ aqueous solution was prepared in the presence of 0.5 wt% PSS. To the solution, 2.7 wt% HMT aqueous solution was added in dropwise under stirring. The produced precipitates were collected and thoroughly washed with ethanol and deionized water. Then, the precipitates slurry was transferred into 0.3 mol/L HNO_3_ aqueous solution and aged for 3 h at 50 °C. After being thoroughly washed in a neural pH value, the PSS-modified TiO_2_ (PSS-TiO_2_) NPs was obtained. XRD pattern in Figure S1 indicated the anatase crystalline of TiO_2_ (JCPDS card No. 21-1272) [40].

**Figure 1:**
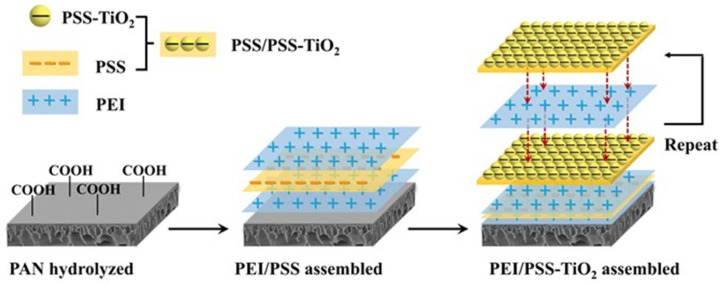
Schematic diagram of fabrication of PE-TiO_2_ nanohybrid membranes.

Next, the PE-TiO_2_ hybrid membrane was fabricated by a self-assembly process. As shown in Figure 1, the hPAN ultrafiltration membrane used as the substrate membrane was immersed into 4.0 g/L of PEI solution for 20 min, followed by being immersed into a 4.0 g/L PSS solution for 20 min to form a cap layer of PAN/(PEI-PSS)_1.0_ membrane (0 bi-layer). Subsequently, the PAN/(PEI/PSS)_1.0_ membrane as a new substrate membrane (SM) was soaked into 5.0 g/L of PEI solution for 20 min, which was followed by being immersed into 5.0 g/L of PSS solution containing 0.20 to 0.30 g/L the prepared PSS-TiO_2_ NPs for 20 min, respectively, to obtain membrane SM/(PEI/PSS-TiO_2_)_1.0_. Such a dipping process was alternatively carried out four times to obtain the membrane (PEI/PSS-TiO_2_)_4.0_. It is worth noting that after each dipping, the redundant polyelectrolytes were removed by rinsing with water. All newly fabricated nanohybrid membranes were irradiated for 0 to 80 min using an ultraviolet (UV) lamp at 365 nm to obtain a superhydrophilic surface.

### 2.3. Membrane Characterization

The membrane surface and cross-sectional morphology were determined by scanning electron microscopy (SEM) and atomic force microscopy (AFM). All membrane samples were dried under a vacuum and sputter-coated with gold before processed to a S-3000N scanning electron microscope (Hitachi Ltd., Tokyo, Japan) for SEM images. AFM images were captured using a Pico Scan TM 2500 Microscope System (Agilent Technologies, Palo Alto, CA, USA) in the tapping mode under ambient conditions. Attenuated total reflectance FTIR spectra (Vertex-70, Bruker, Germany) were used to demonstrate membrane chemical composition. Zeta potential was determined using an Electrokinetic analyzer (Anton Paar, SurPASS, Germany) with a 0.83 mM KCl electrolyte solution and operational pressure of 300 m MPa. Water contact angles were measured using a contact angle analyzer (PSA-100, Bitburg, Germany).

### 2.4. Nanofiltration Performance

The NF performance of the prepared membranes was evaluated using a lab-scale cross-flow NF system, which consisted of plunger pump, pressure gauge, vessel and a circular membrane cell with effective area of 22.4 cm^2^. Prior to measurement, the system was pressurized to 0.6 MPa for 40 min to reach a stable state. The salt or dye solution was used as a feed liquid without adjusting the pH value. Dye concentrations were measured by the absorbance according to the Lambert–Beer law using an ultraviolet-visible spectrophotometer (UV2800, Shanghai, China) at the maximal absorption wavelength of each organic dye. During the nanofiltration process, the concentrate solute was recirculated to the feed vessel, while the permeate liquid was collected in a vessel. The permeance flux (*J*) was calculated by

(1) $$J=\frac{V}{A\Delta t\Delta P}$$

where *V* is the permeate volume; *A* is the membrane effective area; Δ*t* and Δ*P* are operational period and pressure, respectively. And the solute rejection ratio (*R*) by NF membranes was calculated by

(2) $$R=\frac{C_{f}-C_{p}}{C_{f}}\times 100\%$$

where *C_f_* and *C_p_* are solute concentrations in the feed and permeate, respectively.

## 3. Results and Discussions

### 3.1. Self-Assembly of the Charged PE-TiO_2_ Hybrid Membrane

**Figure 2:**
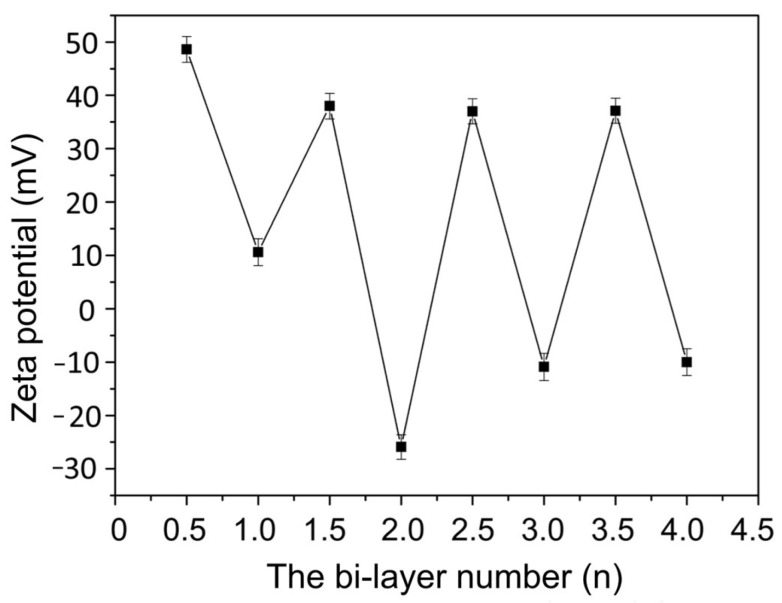
The variation in the zeta potential with the bi-layer number of the membrane.

The (PEI/PSS-TiO_2_)_4.0_ hybrid membrane was fabricated by alternatively self-assembling the positive PEI solution and negative PSS solution containing PSS-TiO_2_ NPs on the SM substrate. To prove the deposition of each layer on the substrate membranes, the surface zeta potentials were tracked using an electrokinetic analyzer. Figure 2 showed the variations in the surface zeta potentials during alternatively assembling PEI and PSS-TiO_2_. When PEI was assembled onto the SM substrate (0 bi-layer), the charge on the membrane surface (0.5 bi-layer) exhibited a positive charge with a zeta potential of +48.7 mV, and then changed to +12.2 mV due to the deposition of the negative PSS-TiO_2_ layer to form the 1.0 bi-layer of the (PEI/PSS-TiO_2_)_1.0_ hybrid membrane. After the deposition of PEI layer, the zeta potential of the membrane (1.5 bi-layer) increased to +38.5 mV. And the zeta potential reversed to −25.9 mV, after assembling PSS-TiO_2_ layer to obtain the (PEI/PSS-TiO_2_)_2.0_ membrane. Then, periodic variations in zeta potential between +37.8 and −10.1 mV appeared for whole multilayer membranes.

**Figure 3:**
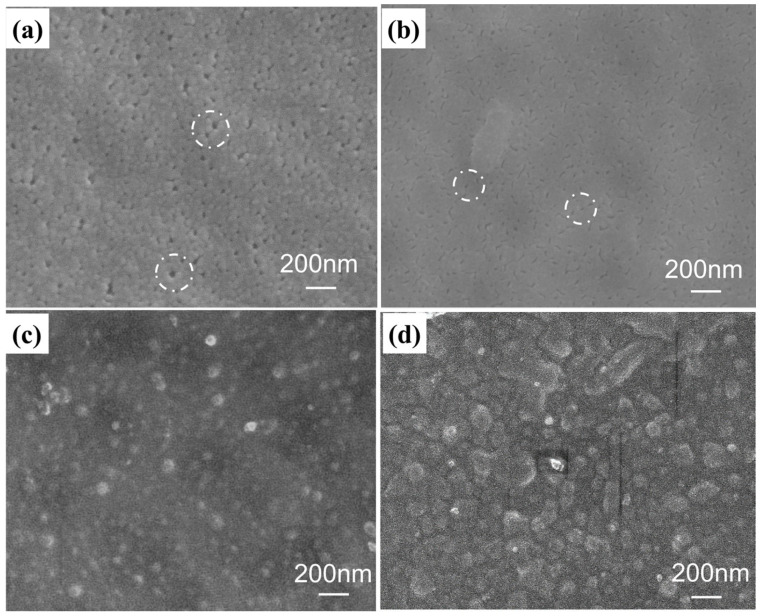
SEM images of (**a**) the hydrolyzed PAN membrane; (**b**) the (PEI/PSS)_1.0_ cap layer; the (PEI/PSS-TiO_2_)_4.0_ membrane with PSS-TiO_2_ NPs concentration of (**c**) 0.2 g/L, and (**d**) 0.3 g/L, respectively (The area in white circle showed pores of the membrane).

The morphologies of different membranes are shown in Figure 3. It was observed that the hPAN membrane (Figure 3a) was porous with a pore size of about 30–50 nm. After the deposition of PEI and PSS to form the (PEI/PSS)_1.0_ cap layers, the surface of the SM substrate (Figure 3b) became smooth, and the pore size obviously decreased to about 15–20 nm. For the alternative deposition of PEI and PSS containing the 0.2 g/L PSS-TiO_2_ NPs solution for four bi-layers, the self-assembled (PEI/PSS-TiO_2_)_4.0_ membrane obviously displayed the TiO_2_ NPs that were uniformly dispersed with a size of about 130 nm (Figure 3c). And the TiO_2_ NPs became some aggregate in the similar membrane with the concentration of PSS-TiO_2_ NPs increasing to 0.3 g/L (Figure 3d). This suggested that the PSS-TiO_2_ were successfully loaded on the membrane surface. However, the higher content of TiO_2_ in the PSS solution might lead to the agglomeration of NPs on the membrane surface.

**Figure 4:**
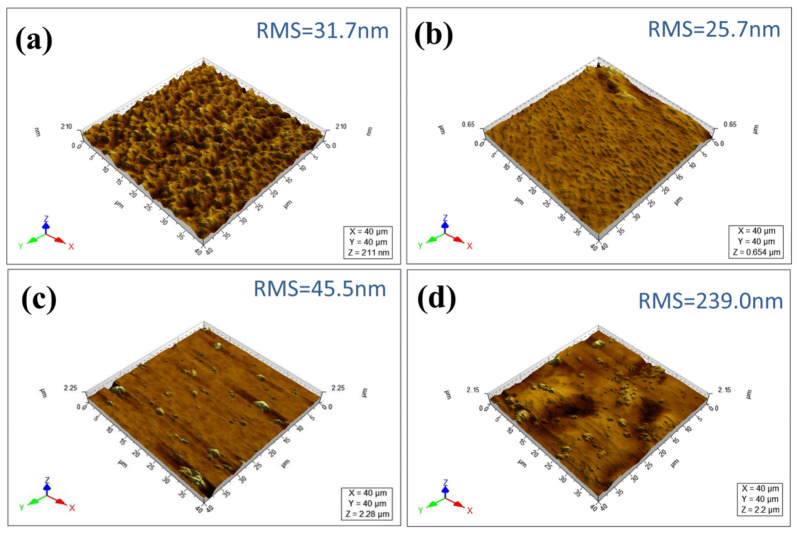
AFM images of (**a**) the hydrolyzed PAN membrane; (**b**) the (PEI/PSS)_1.0_ cap layer; the (PEI/PSS-TiO_2_)_4.0_ membrane with PSS-TiO_2_ NPs concentration of (**c**) 0.2 g/L, and (**d**) 0.3 g/L, respectively.

The surface roughness of the membranes was characterized using AFM shown in Figure 4. It was viewed that the hydrolyzed PAN membrane (Figure 4a) exhibited a roughness of 31.7 nm and the (PEI/PSS)_1.0_ cap layer was a little decreased to 25.7 nm (Figure 4b), indicating that the porous hPAN substrate membrane became smoother after covering the cap layer. This was consistent with the results of SEM images of Figure 3a,b. When the PSS-TiO_2_ NPs in the PSS solution was 0.2 g/L, the membrane roughness increased to 45.5 nm (Figure 4c). With 0.3 g/L of PSS-TiO_2_ NPs in the PSS solution, the roughness of the membrane obviously increased to 239 nm (Figure 4d), due to the aggregation of the TiO_2_ NPs in the membrane (Figure 3). Therefore, the concentration of the PSS-TiO_2_ NPs was properly controlled to less than 0.2 g/L.

**Figure 5:**
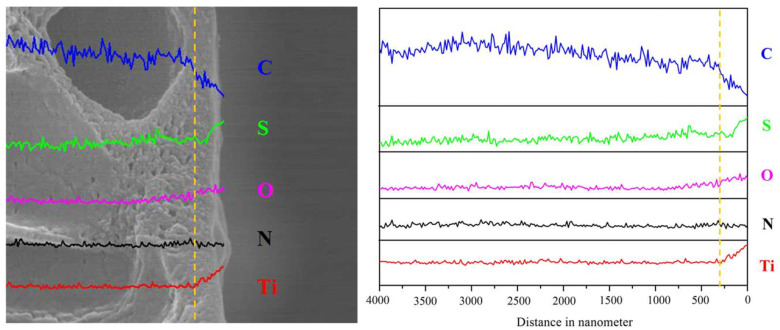
EDX analysis on cross-sectional elements of the (PEI/PSS-TiO_2_)_4.0_ hybrid membrane (the yellowed dashed line indicated the thickness of the membrane through the cross-section).

In order to analyze the thickness of the top selective layer, the changes in elemental composition through the cross-section were also analyzed using EDX shown in Figure 5. It was noted that across the (PEI/PSS-TiO_2_)_4.0_ membrane, the elemental content of carbon (C) content decreased while that of sulfur (S), oxygen (O), and titanium (Ti) increased at a distance of about 250 nm. The elemental amount of nitrogen (N) remained almost unchanged. Since the PAN substrate membrane mainly contained elements of C and N and did not have elements of S, O and Ti, all these elements must arise from the selective PE-TiO_2_ layer. Thus, the thickness of the hybrid membrane with the number (n) of the self-assembled layer was obtained as shown in Figure S2. It indicated that the thickness of the membrane almost linearly increased with the number of the self-assembled layer, similar to our previous membrane without TiO_2_ NPs [39]. The thickness of the (PEI/PSS)_1.0_ cap layers used as SM substrate was about 15 nm. After the deposition of the (PEI/PSS-TiO_2_)_1.0_ membrane, the thickness increased to 87 nm and that of the (PEI/PSS-TiO_2_)_4.0_ membrane was 250 nm with an alternative deposition of the 4.0 layer.

### 3.2. Wettability and Charge of the Membrane Under UV Irradiation

**Figure 6:**
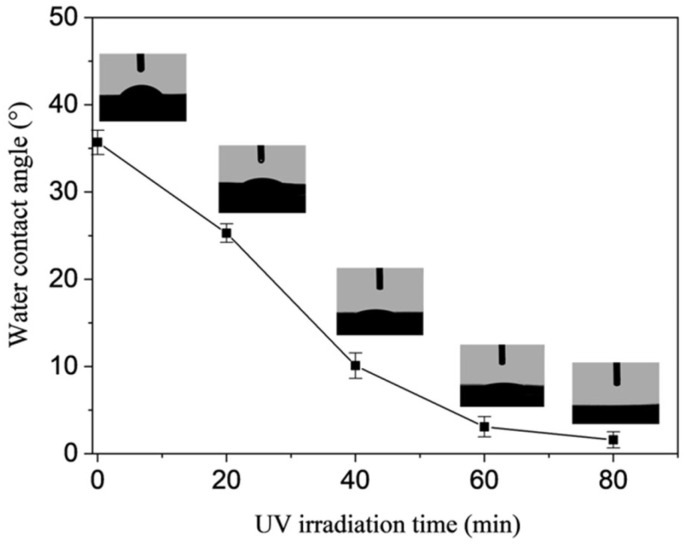
Water contact angles of the (PEI/PSS-TiO_2_)_4.0_ membrane with UV irradiation time.

Due to the existence of the anatase TiO_2_ NPs, the prepared (PEI/PSS-TiO_2_)_4.0_ hybrid membrane showed photocatalysis. As shown in Figure 6, the wettability of the membranes was significantly enhanced with UV irradiation. The initial water contact angle (WCA) of the (PEI/PSS-TiO_2_)_4.0_ hybrid membrane was 35.7°. After UV was irradiated for 20, 40 and 60 min, the WCA decreased to 29.3°, 12.5° and 3.1°, respectively, and the WCA was further decreased to 1.6° after increasing the irradiation time to 80 min, indicating the photocatalytic effect of TiO_2_ in the hybrid membrane that make it superhydrophilicity after ultraviolet irradiation.

Moreover, the zeta potential of the UV irradiated membrane was measured as shown in Figure S3. It was viewed that the initial zeta potential of the (PEI/PSS-TiO_2_)_4.0_ membrane was −10.1 mV, and it gradually decreased to −29.7 mV after UV irradiation for 80 min. The decreased zeta potential upon UV irradiation was probably due to the fact that more hydroxyl groups were produced from the activation of TiO_2_ nanoparticles [41]. This was consistent with the increased hydrophilicity of the membrane with irradiation time shown in Figure 6.

### 3.3. Nanofiltration Performance of the Membranes

**Figure 7:**
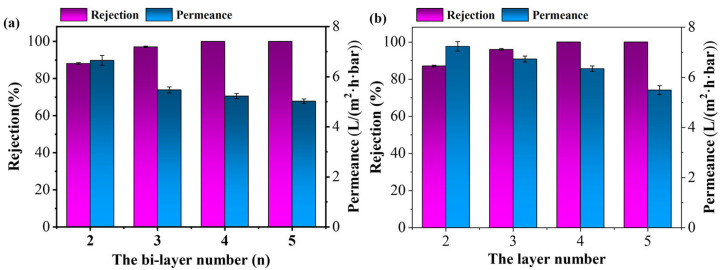
Effect of the layer number on NF of the (PEI/PSS-TiO_2_)_4.0_ membrane (**a**) XO, (**b**) EbT.

The hybrid membrane could impede and reject dyes (such as XO and EbT) while allowing water to pass through, so as to achieve nanofiltration (NF) separation. The NF performance of the membrane with different number of self-assembly layers for retention of XO and EbT was investigated. It was shown in Figure 7 that the rejection of dyes increased with the number of the deposited layers, while the permeance flux declined. When increasing the layer number from 2.0 to 5.0, the rejection of XO and EbT increased from 88.1% and 87.1% to 100%, while the permeance flux of XO solution decreased from 6.7 to 5.0 L/(m^2^·h·bar) and that of EbT decreased from 7.2 to 5.5 L/(m^2^·h·bar). This was due to the thickness of the membrane increasing with the increase in the layer number (Figure S1). This would increase the mass transfer resistance of the membrane and the retention effects [39], leading to an increased rejection and lowered permeance flux. Considering the compromise between rejection and permeance flux, the four number was selected to obtain (PEI/PSS-TiO_2_)_4.0_ membrane, which exhibited 100% rejection to XO and EbT, with permeance flux of 5.2 and 6.4 L/(m^2^·h·bar), respectively.

**Figure 8:**
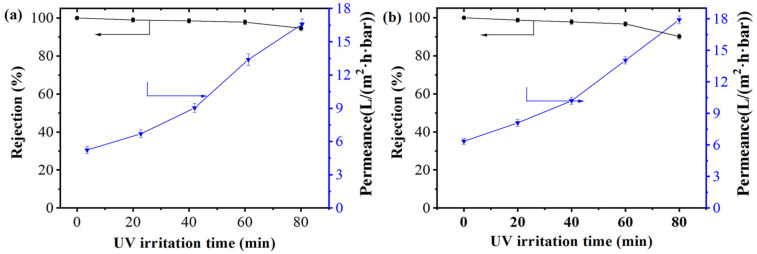
Effect of UV irritation time on NF of the (PEI/PSS-TiO_2_)_4.0_ membrane (**a**) XO, (**b**) EbT.

Due to that the contaminants presented on the membrane could be photocatalytically degraded by anatase TiO_2_, the effect of UV irritation on the nanofiltration of aqueous dye solution was investigated. As shown in Figure 8, with the increase in UV irritation time, the rejection and permeance flux of XO and EbT solution showed different trends between Figure 8a and Figure 8b. The rejection of the two dyes declined gradually, while the permeance flux increased significantly. When the UV irradiation time was 80 min, the rejection of XO and EbT declined to 94.6% and 90.2%, and the permeance flux increased to 16.6 and 17.9 L/(m^2^·h·bar). As seen from Figure S3, although the membrane surface showed more negative charge after UV radiation, the electrostatic effect was not strong enough to balance the rejection decrease caused by some deterioration in the outer layer during ultraviolet radiation. The significant increase in permeation flux was mainly due to the enhanced hydrophilicity of the membrane surface with the extension of UV irradiation time (as shown in Figure 6). Therefore, the suitable UV irradiation time was as 60 min, so as to achieve membrane with permeance flux of 13.4 and 14.0 L/(m^2^·h·bar) and 97.8% and 96.7% rejection to XO and EbT dyes, respectively.

### 3.4. Self-Cleaning of the (PEI/PSS-TiO_2_)_4.0_ Membrane

**Figure 9:**
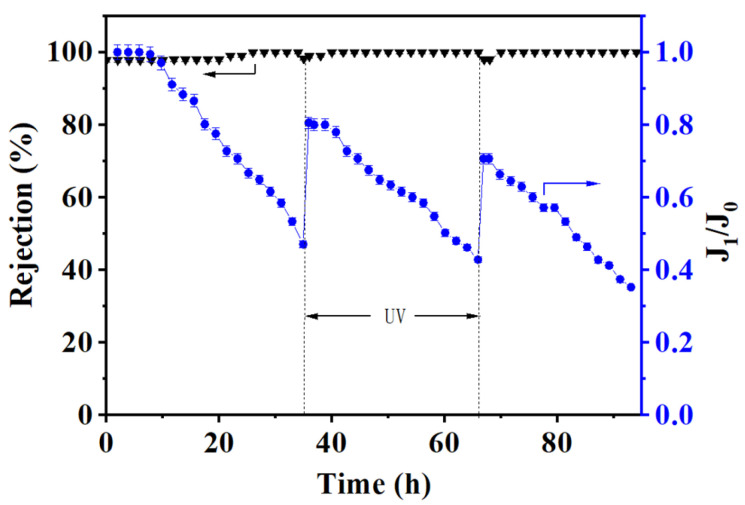
The antifouling property of the (PEI/PSS-TiO_2_)_4.0_ hybrid membrane for NF of XO solution and UV irritated for 30 min.

The self-cleaning property of the (PEI/PSS-TiO_2_)_4.0_ membrane was achieved via UV radiation to alleviate membrane fouling. During the NF separation of XO solution, when the permeance flux declined to half, the membrane was radiated by UV light for 30 min. As shown in Figure 9, the permeance flux of the membrane declined to 50% when running for 35 h. After UV irradiation for 30 min, the permeance flux recovered to 80.5%. Continuing the operation, the permeance flux decreased again to 43% in a period of 70 h and then recovered to 70.7% after 30 min UV irradiation. Finally, the permeance flux declined to 35.2% after 95 h. During each operation cycle, the decrease in the permeance flux was mainly due to the membrane fouling caused by the rejected XO dye. The photocatalytic effect of the anatase TiO_2_ NPs in the membrane could degrade some dyes leading to flux recovery. Owing to the low content of TiO_2_ NPs in the membrane, the photocatalytic effect seemed not so ideal that the permeance recovery was about 80.6% after 30 min UV irradiation. Yet, the dye rejection remained at about 97.8% to 100%, whether the membrane was irritated by UV light or suffered membrane fouling, indicating the outer layer of the membrane was much stable. The reason was that TiO_2_ nanoparticles acted as space holders among the polyelectrolytes bi-layers, avoiding shrinkage or deterioration of the PEs layer to some extent upon UV irradiation [42]. Moreover, the comparison on the photocatalytic self-cleaning performance of the (PEI/PSS-TiO_2_)_4.0_ membrane with other reported ones was shown in Table S2. It indicated that compared with others, the hybrid membrane exhibited a compromised permeance recovery, owing to the low content of TiO_2_ NPs.

**Figure 10:**
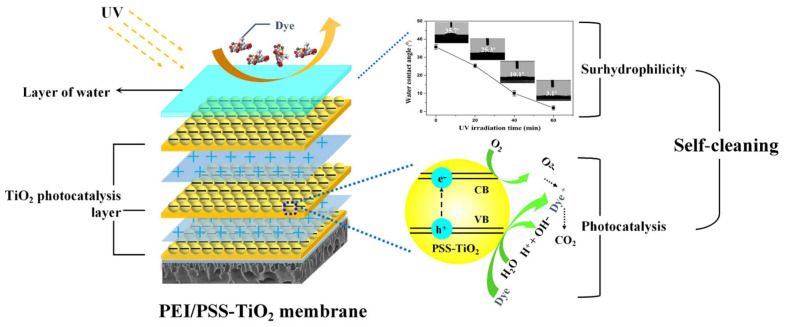
Schematic of the self-cleaning mechanism of the (PEI/PSS-TiO_2_)_4.0_ membrane.

It has demonstrated that the prepared PSS-TiO_2_ NPs exhibited good photocatalytic effect [40], which can lead to the decomposition and removal of even organic contaminants under ultraviolet irradiation [17,42]. The (PEI/PSS-TiO_2_)_4.0_ hybrid membrane showed photoinduced superhydrophilicity (Figure 6). The mechanism of this photocatalytic self-cleaning and superhydrophilicity was depicted as in Figure 10. On one hand, the superhydrophilicity was due to the adsorption of water molecules in the air on the surface of hydrophilic membrane, which could bring OH groups and enhance the photocatalytic performance under UV irradiation. On the other hand, the photocatalytic degraded organic pollutants could increase the surface hydrophilicity [40]. Due to the PSS-TiO_2_ NPs existing in the outer layer of the membrane could chemisorb H_2_O molecules, which could further adsorb more water molecules by van der Waals forces and hydrogen bonds [43]. The formed hydrophilic layer could efficiently prevent the contaminants from contacting the surface and enhance the anti-fouling property. Thus, the pollutants on the surface could be easily detached from the surface under the action of water. These results suggested the self-cleaning (PEI/PSS-TiO_2_)_4.0_ membrane was achieved by the synergetic action of superhydrophilicity and photocatalysis.

## 4. Conclusions

In conclusion, the negatively charged (PEI/PSS-TiO_2_)_4.0_ membrane was fabricated by the self-assembly of polyelectrolyte containing PSS-modified anatase TiO_2_ nanoparticles. The water contact angle of the membrane could be decreased from 35.7° to 1.6° under UV irradiation, leading to the superhydrophilic surface. The optimal (PEI/PSS-TiO_2_)_4.0_ membrane exhibited 100% rejection to XO and EbT, with permeance flux of 5.2 and 6.4 L/(m^2^·h·bar), respectively. The permeance flux could be further increased to 13.4 and 14.0 L/(m^2^·h·bar), and the rejection remained at 97.8% and 96.7% after UV irradiation for 60 min. Owing to the low content of TiO_2_ NPs photocatalytic effect under UV irradiation, the fabricated hybrid membrane exhibited a compromised permeance recovery of about 80.6%. Thus, the subsequent work will focus on the loaded TiO_2_ NPs into the membrane and its antifouling property.

## Data Availability

The original contributions presented in this study are included in the article/Supplementary Materials. Further inquiries can be directed to the corresponding authors.

## Supplementary Materials

The following supporting information can be downloaded at: https://www.mdpi.com/article/10.3390/membranes15060179/s1, Figure S1: The XRD pattern of the prepared PSS-TiO_2_ nanoparticles; Figure S2: The variation of the membrane thickness with number of the self-assembled layer; Figure S3: The variation of the zeta potential of the membrane with UV irradiation time; Table S1: Physical properties of different dyes; Table S2: Performance comparison of the membrane in this work with others. References [44,45,46,47] are cited in the Supplementary Materials.

## Abbreviations

NF	Nanofiltration
PAN	polyacrylonitrile
PEI	Poly(ethyleneimine)
PSS	poly(sodium4-styrenesulfonate)
XO	Xylenol orange
EbT	eriochrome black T
SM	new substrate membrane
WCA	water contact angle
