# Supplementary material for: The Charged Superhydrophilic Polyelectrolyte/TiO2 Nanofiltration Membrane for Self-Cleaning and Separation Performance
Source: Membranes (Basel). 2025 Jun 12;15(6):179. doi: 10.3390/membranes15060179 (PMC12195473; doi:10.3390/membranes15060179)
Supplement: Supplementary file 1 [file membranes-15-00179-s001.zip › membranes-3644289-supplementary.pdf]

Article

The charged superhdrophilic polyelectrolyte/TiO_2 nanofiltration membrane for self-cleaning and separation performance

Weiliang Gu [1,2], Lei Han [3], Ye Li [1], Jiayi Wang [1], Haihong Yan [4,*], Zhenping Qin [1] and Hongxia Guo [1,*]

1 State Key Laboratory of Materials Low-Carbon Recycling, College of Materials Science and Engineering, Beijing University of Technology, Beijing 100124, China; guweiliang@emails.bjut.edu.cn (W.G.); limingye@emails.bjut.edu.cn (Y.L.); jiayiwang@emails.bjut.edu.cn (J.W.); zhenpingq@bjut.edu.cn (Z.Q.)
2 Beijing Nansheng Technology Co., Ltd., Beijing 102601, China
3 Xinkai Environment Investment Co., Ltd., Beijing 101101, China; hanlei726@126.com
4 State Key Laboratory of Environmental Criteria and Risk Assessment, Research Center of Environmental Pollution Control Engineering Technology, Chinese Research Academy of Environmental Sciences, Beijing 100012, China
* Correspondence: yanhh@craes.org.cn (H.Y.); hxguo@bjut.edu.cn (H.G.)

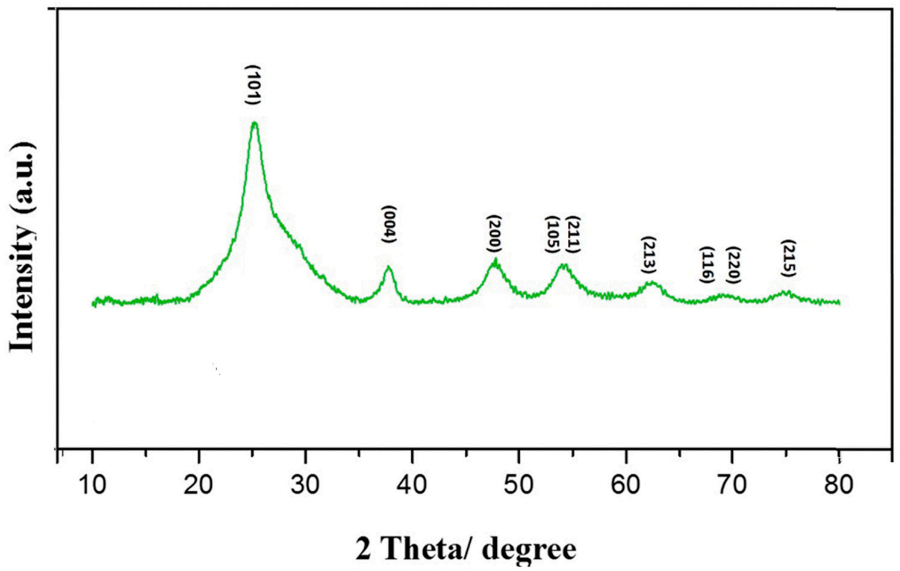

Figure S1. The XRD pattern of the prepared PSS-TiO_2 nanoparticles

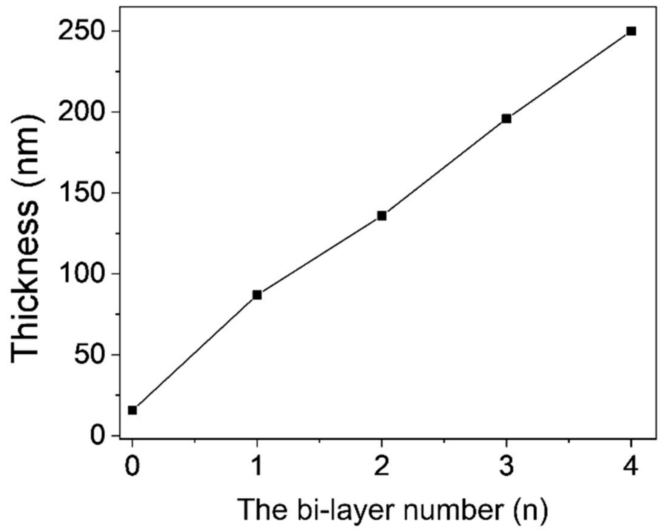

Figure S2 The variation of the membrane thickness with number of the self-assembled layer

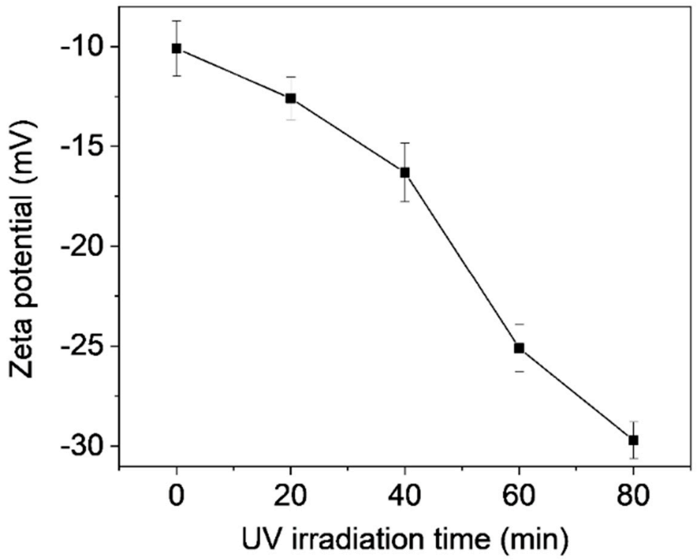

Figure S3 The variation of the zeta potential of the membrane with UV irradiation time

Table S1. Physical properties of different dyes

Dyes	Eriochrome black T	Xylenol orange
Formula	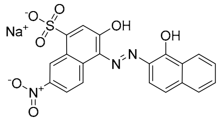	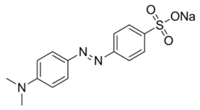
Molecular weight(g/mol)	461.4	716.6
Wavelength (nm)	336	432
Charge	negative	negative

Table S2. Performance comparison of the membrane in this work with others.

Membrane	TiO_2 content (wt%)	initial performance		after UV irradiation		Ref.
		Type of solute, Rejection (%)	Permeance flux (LMH)	Cycle time, rejection (%)	Permeance recovery (%)	
PA/TiO_2	5.0	$MgSO_4$, 95	9.1, 0.6MPa	---	----	1
PA/TiO_2	1.5	CR, 99.7<br>MB, 99.1	14.32 bar	1.5 h<br>99.0	98.6	2
PA CQD/TiO_2	--	MB, 99.1<br>CR, 99.8	67.22 bar	5.0 h<br>98.0	98.5	3
PEI/GO-TiO_2	0.6	MB, 79	1.67	100 min<br>58.8	54	4
GO-TiO_2	25	MB, 99.3<br>RhB, 99.4	3.6	24 h<br>99	86.1	5
GO/C_3N_4/TiO_2-CNT	--	MO, 100	5.4 bar	150 min<br>95	77.8	6
**This work**	0.2	XO,100<br>EbT, 100	5.2 bar<br>6.4 bar	35 h<br>XO:94.6<br>EbT:90.2	80.6	This work

Note: Congo red (CR), methyl blue (MB), Rhodamine B (RhB), Methyl Orange (MO), Xylenol orange (XO), Eriochrome black T (EbT)

References

1. Lee, H., S.; Im., S., J.; Kim, J., H.; Kim, H., J.; Kim, J., P.; Min, B., R. Polyamide thin-film nanofiltration membranes containing TiO_2 nanoparticles. Desalination **2008**, *219*, 48–56

2. Xie, Y.; Cheng, L.; Liu, F.; Li, J. TiO_2-Decorated Photocatalytic Nanofiltration Membranes for Enhanced Self-Cleaning and Separation Performance. ACS EST Water **2023**, *3*, 3418–3427
3. Mi, Y.; Wang, N.; Fang, X.; Cao, J.; Tao, M,; Cao, Z. Interfacial Polymerization Nanofiltration Membrane with Visible Light Photocatalytic Self-cleaning Performance By Incorporation of CQD/TiO_2. Sep. Purif. Technol. **2021**, *277*, 119500
4. Yan, X.; Huo, L.; Ma, C.; Lu, J. Layer-by-layer assembly of graphene oxide-TiO_2 membranes forenhanced photocatalytic and self-cleaning performance. Process Saf. Environ. **2019**, *130*, 257–264
5. Liu, Y.; Yu, Z.; Peng, Y.; Shao, L.; Li, X.; Zeng, H. A novel photocatalytic self-cleaning TiO_2 nanorods inserted graphene oxide based nanofiltration membrane. Chem. Phys. Lett. **2020**,*749*, 137424
6. Zhang, Q.; Chen, S.; Fan, X.; Zhang, H.; Yu, H.; Quan, X. A multifunctional graphene-based nanofiltration membrane under photoassistance for enhanced water treatment based on layer-by-layer sieving. Appl. Cat. B: Environ. **2018**, *224*, 204–213
